# Myocardial iron quantification using T2* and native T1mapping - a 250 patient study

**DOI:** 10.1186/1532-429X-17-S1-P312

**Published:** 2015-02-03

**Authors:** Amna Abdel-Gadir, Daniel Sado, Stuart Murch, Viviana Maestrini, Stefania Rosmini, Thomas A Treibel, Marianna Fontana, Heerajnarain Bulluck, Stefan K Piechnik, Charlotte Manisty, Anna S Herrey, John Malcolm Walker, John Porter, James Moon

**Affiliations:** 1 The Heart Hospital Imaging Centre, UCLH, London, UK; 2 University College London, London, UK; 3 John Radcliffe Hospital, Oxford, UK

## Background

The management of iron overload has been transformed by the use of T2* as a surrogate marker of cardiac iron loading. The presence of iron however not only changes T2*, but also T2 and T1. Recent advances make T1 mapping a possible complementary technique to T2*. Preliminary data are encouraging, but the relative advantages and disadvantages and optimal mathematical model for the relationship between T1 and T2* remain unknown.

## Methods

This was a single centre prospective study of 250 patients (age 37 ± 13 years) referred with potential iron overload for T2* assessment, with 50 healthy volunteers (age 44 ± 11 years) as a reference comparison group. Each participant underwent short axis septal T2* (standard Siemens sequence using 8 different TE) at 1.5T and in addition myocardial T1 mapping (ShMOLLI sequence).

## Results

### Image quality

27% of patients required more than one T2* acquisition to obtain optimal images for analysis compared with 12% for T1 mapping. ShMOLLI images were uninterpretable in 2 patients due to the presence of an MRI conditional pacemaker, and the positioning of a PORT-A-CATH implantable venous access system. It is known that some patients were unable to complete T2* sequences (21 heart beat scan), but these were not captured by the inclusion criteria of the study.

**Figure 1:**
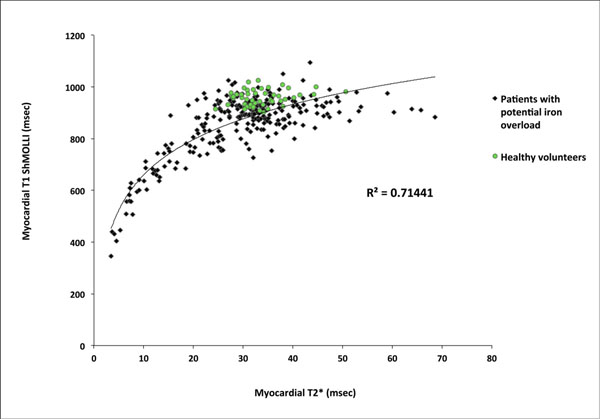
Correlation between myocardial T2* and native T1 mapping measurements in patients (black dots) and healthy volunteers (green dots)

**Figure 2:**
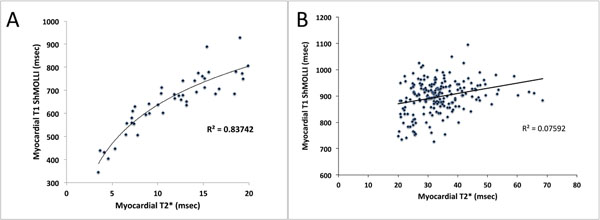
A: Correlation between myocardial T2* LESS than 20ms and native T1 mapping. B: Correlation between myocardial T2* GREATER than 20ms and native T1 mapping

There was an exponential relationship between T1 and T2* across all patients and healthy volunteers (R^2^=0.71, p<0.001, figure 1). This was composed of a tight curvefit below T2* of 20ms (R^2^=0.83, p<0.001, figure 2A) and almost no correlation above 20ms (R^2^=0.07, figure 2B). The lower limit of normal (2SD below the mean) T1 from the healthy volunteers was 895ms. In patients with T2* above 20ms, T1 was normal in 55%, high in 1% and low in 44%.

The derived equivalent T1 cutpoints for published T2* cutoffs for mild, moderate and severe iron were 20ms (846ms); 14ms (705ms) and 10ms (636ms). If T1 is accurate for iron, then using the lower limit of normal for T1 (895ms) would suggest that the normal limit for T2* would be 29ms. However, of the 50 healthy volunteers, 8 (16%) had T2*s lower than this, possibly due to relatively lower precision of T2* measurements.

## Conclusions

In potential cardiac iron overload, not all patients manage good image quality on the first breath-hold with either technique. Measured myocardial T1 and T2* are best modelled using an exponential curve fit but only correlate below a T2* of 20ms. T1 data suggests that the lower limit of normal T2* should be 29ms and thus far more patients have myocardial iron than is currently recognised - but such a high cutpoint for T2* would generate a poor specificity.

## Funding

AAG is supported by a research grant from the Rosetrees Trust.

